# The relationship among body image, psychological distress, and quality of life in young breast cancer patients: a cross-sectional study

**DOI:** 10.3389/fpsyg.2024.1411647

**Published:** 2024-08-21

**Authors:** Hongmei Yao, Meidi Xiong, Yuping Cheng, Qingyuan Zhang, Ying Luo, Xiegang Ding, Chunhua Zhang

**Affiliations:** ^1^Nursing Department, Zhongnan Hospital of Wuhan University, Wuhan, China; ^2^Medical Department, Yangtze University, Jingzhou, China; ^3^Beihu Community Health Service Center, Wuhan, China

**Keywords:** young breast cancer patient, body image, quality of life, psychological distress, medical coping

## Abstract

**Purpose:**

The aim of this study is to explore the interrelationships among body image perception, levels of psychological distress, and the quality of life (QOL) experienced by young breast cancer patients.

**Methods:**

This study analyzed data from 339 young female breast cancer patients aged between 18 and 40 years (mean age was 33.47 years) from August 2023 to February 2024. Data on demographic characteristics, psychological distress, body image, medical coping, and QOL of young breast cancer patients were collected. Psychological distress, body image, medical coping, and QOL were measured using the Distress Thermometer (DT), Hospital Anxiety and Depression Scale (HADS), Body Image Scale (BIS), Medical Coping Modes Questionnaire (MCMQ), and Functional Assessment of Cancer Therapy-Breast (FACT-B), respectively. Multiple regression analysis was conducted to examine factors influencing QOL.

**Results:**

After adjusting for covariates, significant predictors of QOL in young survivors included psychological distress (*β* = −3.125; *p* = 0.002), anxiety and depression (*β* = −4.31; *p* < 0.001), cognitive dimension of body image (*β* = −0.218; *p* = 0.027), behavioral dimension of body image (*β* = 0.579; *p* = 0.047), and confrontational dimension of medical coping (*β* = −0.124; *p* = 0.01).

**Conclusion:**

The findings suggest that higher levels of body image concerns and psychological distress are associated with poorer QOL among young female breast cancer patients. Furthermore, breast cancer patients facing with more positive medical coping strategies predicted a higher QOL.

## 1 Introduction

In 2021, the World Health Organization reported that breast cancer is the most prevalent cancer worldwide (Sung et al., 2021). In Western nations, women typically experience the highest incidence after the age of 60 (Fitzmaurice and Global Burden of Disease Cancer Collaboration, 2018), whereas in Asia, the peak age is in the 40s. Notably, 20% of breast cancer cases occur in younger women (Fitzmaurice and Global Burden of Disease Cancer Collaboration, 2018). In young breast cancer (YBC) patients, issues related to body image are a continual challenge (Chopra et al., 2021). This is exacerbated by the more aggressive treatments typically received (Perrault et al., 2020), which can lead to physical changes such as breast deformities, hair loss, skin alterations, weight gain, and other appearance-related issues (Lovelace et al., 2019). These changes significantly contribute to body image disturbances experienced by YBC patients (Przezdziecki et al., 2016). Research indicates that in the United States, up to 35 to 40% of young breast cancer patients choose to undergo bilateral mastectomy, a percentage that has been steadily rising (Kurian et al., 2014; Katz et al., 2018). Therefore, emphasizing body image is essential (Davis et al., 2020), as it can stimulate the development of unhealthy behavioral patterns and a spiral of negative emotions, ultimately impacting the quality of life (QoL) of women following breast cancer diagnosis (Paterson et al., 2016). A study involving 406 recently operated-on female breast cancer survivors indicated that those with poorer body image reported lower breast cancer-specific and overall QoL (Zhou et al., 2020). A study involving 4,567 female cancer survivors found significant associations between body image, appearance, physical activity, and symptoms of anxiety and depression (Zhang et al., 2021).

In addition to the challenges posed by the disease itself, YBC patients often contend with a variety of life issues, including fertility concerns, sexual function, body image, fear of cancer recurrence (Kowalczyk et al., 2019; Morales-Sánchez et al., 2021), as well as challenges in marital relationships, financial burdens, and social interactions. Therefore, YBC patients also experience higher rates of psychological distress (Howard-Anderson et al., 2012). Psychological distress encompasses the psychological discomfort, suffering, or pain that individuals experience, which not only diminishes their ability to cope with cancer (Waller et al., 2013), affecting their readiness for treatment and adversely impacting treatment outcomes and prognosis, but also significantly reduces the QoL of YBC patients. A study (Zhang et al., 2022) on breast cancer summarized research hotspots, revealing “breast cancer” and “health-related quality of life” as the top two keywords. This underscores that QoL remains the foremost outcome of interest for researchers. QoL refers to individuals’ perception of their life, encompassing primarily their physical, psychological, and social functioning. It serves as a vital indicator for measuring adult health outcomes (Kastora et al., 2023).

Research reports indicate that interpersonal relationships, psychological distress, body image, and sexual issues are closely related to different aspects of QoL among YBC patients (Miaja et al., 2017). Dissatisfaction with one’s body image not only exacerbates psychological distress but also impacts breast cancer patients’ ability to cope with medical challenges, such as understanding the disease, actively participating in treatment plans, and adhering to medical advice (Zhu et al., 2022). Medical coping patterns, also known as medical coping strategies, refer to how patients respond to stressful events, influenced by their personal characteristics and experiences. These patterns encompass three dimensions: confrontation, avoidance, and resignation (de Wit et al., 2019). Positive coping with body image implies accurate body perceptions, positive thoughts, and feelings, primarily demonstrated through adaptive actions or health-promoting behaviors (Torres et al., 2022). Specifically, this can help breast cancer patients gain more confidence, reduce self-criticism, enhance self-acceptance, and better withstand external influences such as criticism from others and societal or cultural pressures (Brunet et al., 2022). Therefore, it is important to encourage young breast cancer patients to discover attitudes and coping strategies that work best for them. This not only helps them effectively manage their illness and adapt to lifestyle changes from treatment but also enhances their overall QoL.

To date, research on the relationship between psychological distress, body image, and QOL in young breast cancer patients remains insufficient. This study aims to assess the psychological distress experienced by young breast cancer patients during treatment and recovery, including anxiety and depression, and how this distress impacts their QOL. Additionally, the study will investigate changes in body image among these survivors, focusing on the effects on their self-perception, emotions, and behaviors. Furthermore, it will explore how different medical coping strategies influence survivors’ ability to face the challenges of the disease and subsequently affect their QOL. Based on a literature review, we propose three hypotheses:

Hypothesis 1: Each dimension of body image in young breast cancer patients is negatively correlated with their QOL.

Hypothesis 2: Psychological distress is negatively correlated with QOL.

Hypothesis 3: Positive medical coping strategies are positively correlated with QOL.

## 2 Methods

### 2.1 Study design

This cross-sectional survey was conducted at the Department of Breast Surgery, Zhongnan Hospital of Wuhan University. A total of 339 breast cancer patients participated in the study from August 2023 to February 2024. The study received approval from the Institutional Review Board of the hospital (Protocol [2,023,049 K]).

### 2.2 Participants

This study included female breast cancer patients who met the following criteria: diagnosed with breast cancer, aged between 18 and 40 years, capable of fluent verbal expression or completing questionnaires, and willing to participate in the study. Participants with pre-existing mental or cognitive disorders before breast cancer diagnosis were excluded, as the study focused on the impact of breast cancer itself on psychological distress, body image, and medical coping strategies. The presence of mental or cognitive disorders was assessed through comprehensive medical history, initial diagnostic evaluations, and self-disclosure during participant recruitment, ensuring internal validity and accuracy of the study.

A total of 378 eligible female participants were enrolled in this study. Among them, 339 valid questionnaires were utilized, resulting in an effective response rate of 89.68%. These participants were recruited across different stages of breast cancer treatment, including those currently undergoing treatment, post-surgery, and during follow-up. The average time since breast cancer diagnosis to participation was 1.1 years. Questionnaires that were incomplete, inconsistent in answers, or missing key variable data were considered invalid and excluded from data analysis, ensuring the integrity and reliability of the final analysis.

### 2.3 Measures

#### 2.3.1 Demographic characteristics questionnaire

Demographic data, encompassing variables such as Age, number of children, religion, educational Level, careers, work status, *per capita* monthly household income, medical cost, self-managed ability, surgical form, cancer stage, hair loss types, mode of treatment, number of chemotherapy cycles and number of radiation cycles, were collected.

#### 2.3.2 Body image

Body image was assessed using a 10-item Body Image Scale (Hopwood et al., 2001), which utilizes a 3-point Likert scale ranging from 1 (not at all) to 3 (very much) to evaluate dissatisfaction with cancer- or treatment-related appearance changes. It comprises three dimensions: body image emotions, behaviors, and cognitions. A higher total score indicates lower body image levels and more severe body image disturbances. The scale showed high reliability (Cronbach’s alpha 0.93) and good clinical validity based on response prevalence, discriminant validity (*p* < 0.0001, Mann ± Whitney test), sensitivity to change (*p* < 0.001, Wilcoxon signed ranks test) and consistency of scores from different breast cancer treatment centers. The Chinese version of the BIS has been tested among cancer survivors and is psychometrically sound (Jiayan and Changlian, 2019). In this study, the Cronbach’s alpha value was 0.85.

#### 2.3.3 Quality of life

The QOL of breast cancer patients was assessed using the 36-item Functional Assessment of Cancer Therapy-Breast (Cella et al., 1993). Cronbach’s alpha value of the original scale was 0.69–0.82, indicating good reliability. Each item of this tool is rated on a 5-point scale from 0 (not at all) to 4 (very much), encompassing five dimensions: physical well-being, social/family well-being, emotional well-being, functional well-being, and breast cancer-specific concerns. A higher total score indicates a higher QOL. The Chinese version (Chonghua et al., 2003) of the FACT-B has been used among cancer survivors with high reliability and validity. In this study, Cronbach’s alpha value was 0.82.

#### 2.3.4 Medical coping

The assessment of medical coping strategies in breast cancer patients is conducted using the sophisticated 20-item Medical Coping Modes Questionnaire (Feifel, 1987). This instrument employs a 4-point Likert scale, ranging from 1 (infrequent) to 4 (very frequent), to evaluate the coping levels of cancer survivors when confronting medical issues or treatments. It encompasses three dimensions: facing, avoiding, and yielding. Higher score in each dimension signifies a more frequent application of the respective coping strategy by the patient. Presently, this questionnaire is extensively employed across diverse patient populations in China (Xiaohong and Qianjin, 2000), encompassing individuals with conditions such as cancer, diabetes, and liver diseases, demonstrating notable reliability and validity. Cronbach’s alpha value of the original scale was 0.66–070. In the context of this study, the Cronbach’s alpha coefficient was calculated to be 0.82.

#### 2.3.5 Psychological distress

Psychological distress among breast cancer patients is evaluated through the utilization of two assessment tools: the Hospital Anxiety and Depression Scale (Zigmond and Snaith, 1983) and the Distress Thermometer (Roth et al., 1998). The Hospital Anxiety and Depression Scale consists of 14 items, utilizing a 4-point Likert scale ranging from 0 (absent) to 3 (persistent) to evaluate the extent of anxiety and depression in breast cancer patients (Zigmond and Snaith, 1983). It encompasses two dimensions, anxiety and depression. Higher scores indicate more severe symptoms described by the respective dimension. The original scale uses spearman to test the internal consistency of the two subscales, for depression r = 0.70, for anxiety r = 0.74. In this study, the Cronbach’s alpha value was 0.91.

The Distress Thermometer comprises a single-item screening tool along with a Problem List (PL) questionnaire (Roth et al., 1998). The PL incorporates practical, interpersonal, emotional, physical, and spiritual/religious concerns. It employs a binary ‘yes’ or ‘no’ assessment to identify the presence of psychological distress in survivors within specific dimensions. Integrated with the PL, survivors utilize a single-item screening tool to quantify the degree of psychological distress on a numeric scale ranging from 0 to 10, encompassing 11 levels (0 represents no distress, 1–3 denotes mild distress, 4–6 indicates moderate distress, 7–9 signifies severe distress, and 10 reflects extreme distress). Survivors are guided to rate their recent psychological distress over the past week, with a DT score of ≥4 considered clinically significant.

## 3 Data analysis

Descriptive analysis was utilized to discern the demographic and lifestyle features of participants, alongside the distribution of data pertaining to psychological distress, body image, medical coping, and QOL. These characteristics encompass a spectrum of continuous and categorical variables. Continuous variables are presented as mean values with standard error (SE), with age being among them. Categorical variables are represented in numerical values and percentages, covering factors such as the highest number of children, religious affiliation, education level, employment status, work status, average monthly household income, medical expenses, self-care ability, surgical approach, disease stage, type of hair loss, treatment method, chemotherapy and radiotherapy sessions which were analyzed using chi-square tests. Individual traits were acknowledged as potential covariates. We utilized univariate (simple) linear regression to identify potential factors or covariates, encompassing demographic, disease-related factors, and independent variables (Table 1). The independent variables included BIS scores, MCMQ scores, FACT-B scores, HADS scores, DT scores, as well as scores for each dimension of each scale (Table 2). Furthermore, we employed Multivariate linear regression analysis to examine whether the independent variables predict FACT-B scores in breast cancer patients after adjusting for covariates (Table 3). These independent variables are variables with *p* values <0.10 in univariate regression analyses treated as covariates including Educational Level, Careers, Work status, *Per capita* monthly household income, Medical cost, Self-managed ability, Surgical form, Cancer stage, Hair Loss Types, Mode of treatment, Number of chemotherapy cycles, and Number of radiation cycles. The internal consistency and reliability of each instrument were assessed using Cronbach’s alpha coefficient. Missing data were excluded from all analyses. All statistical tests were significant at the *p* < 0.05 level. Statistical analysis was conducted using SPSS 27.

**Table 1:** Characteristics of the study participants, and the association with the QOL for breast cancer patients.

Characteristic	Mean or *n*	SE or %	FACT-B score median (25th to 75th IQR)	X^2^	p (post hoc)
Age (yrs)	33.47	–	–	2.05	0.38
Number of children					
0	7	2.06%	70.5(68,.)	4.05	0.26
1	155	45.72%	71(69,73.75)		
2	132	38.94%	69(65,74)		
≥3	45	13.27%	72(68.75,73.25)		
Religion					
Yes	57	16.81%	69(66,73)	0.07	0.79
No	282	83.19%	71(68,74)		
Educational Level					
Primary school	46	13.57%	70(67,74)	32.08	<0.001**
Secondary school	100	29.50%	69(67,72)		
High school	74	21.83%	72(65.5,74)		
Diploma	46	13.57%	73(70.5,76.75)		
Bachelor’s degree or higher	73	21.53%	71(58.5,77)		
Careers					
Sole trader	49	14.45%	78(69,80)	64.88	<0.001**
Teacher	29	8.55%	70.5(69.25,73)		
Medical personnel	27	7.96%	76(70,.)		
Worker	123	36.28%	72(70,74)		
National public servant	21	6.19%	72(71.25,72)		
Peasant	51	15.04%	66(62,68)		
Other occupations	39	11.50%	71(69.25,74)		
Work status					
Currently working	72	21.24%	69(63.25,79.25)	75.80	<0.001**
Retire	98	28.91%	70(66.25,72.75)		
Sick leave	70	20.65%	70(67,74)		
Leave office	17	5.01%	70(65,73)		
Other	82	24.19%	71(68.5,74)		
*Per capita* monthly household income (yuan)					
<1,000	41	12.09%	68(65.75,70)	99.18	<0.001**
1,000–2,999	70	20.65%	72(66,74)		
3,000–4,999	133	39.23%	71(69,73)		
5,000–10,000	68	20.06%	80(72,85)		
>10,000	27	7.96%	–		
Medical cost					
Medical insurance	257	75.81%	71(69,74)	44.33	<0.001**
Free medical service	30	8.85%	72(71,74)		
Own expense	34	10.03%	66(62,73)		
Other	18	5.31%	68(64,68)		
Self-managed ability					
Full self-care	138	40.71%	72(71,78)	111.34	<0.001**
Partially self-managed	88	25.96%	71(69,73)		
Unable take care of oneself	113	33.33%	68(64,72)		
Surgical form					
Mastectomy	159	46.90%	70(67,73)	133.29	<0.001**
Lumpectomy	180	53.10%	76.5(69,80.5)		
Cancer stage					
Stage I	65	19.17%	–	135.47	<0.001**
Stage II	149	43.95%	70(65,71.5)		
Stage III	118	34.81%	70(67,74)		
Unclear	7	2.06%	–		
Hair loss types					
Patchy hair loss	38	11.21%	–	111.71	<0.001**
Localized hair loss	36	10.62%	–		
Diffuse hair loss	101	29.79%	69(66,72.5)		
Total hair loss	119	35.10%	72(68,74)		
No hair loss	45	13.27%	–		
Mode of treatment					
Surgery	44	12.98%	–	134.59	<0.001**
Chemotherapy and surgery	145	42.77%	–		
Chemotherapy, surgery and radiation	109	32.15%	70(67,73.5)		
Chemotherapy	41	12.09%	–		
Number of chemotherapy cycles					
4	92	27.14%	81(78,84.25)	152.10	<0.001**
8	203	59.88%	70(67,73)		
Number of radiation cycles					
25–30	32	9.44%	74(74,76)	19.52	<0.001**
30–35	77	22.71%	69(66,71)		

Data are mean ± standard error (SE) or n (%); BMI, body mass index. **p < 0.1: The association between personal characteristics and the QOL for breast cancer patients was examined by a univariate regression analyses to be treated as covariates in the multivariate regression model.

**Table 2:** Scores and distribution of QoL, body image, psychological distress and medical coping skills.

Project	Mean or n	SE or %
QOL (FACT-B)		
Physical well-being^a^	14.04	6.51
Social/Family well-being^a^	15.32	7.54
Emotional well-being^a^	12.78	5.31
Functional well-being^a^	14.98	6.84
Breast cancer-specific well-being ^a^	18.70	8.04
Total score^a^	75.75	7.39
Body image(BIS)		
Affective dimension^a^	5.74	2.96
Cognitive dimension^a^	5.74	2.91
Behavioral dimension^a^	2.97	1.66
Total score^a^	14.45	7.18
Psychological distress(DT and HADS)		
Psychological distress thermometer(DT)		
<4^b^	100.00	0.29
≥4^b^	239.00	0.71
Anxiety(HADS-A) ^a^	8.28	4.71
Depression(HADS-D)^a^	8.44	4.89
Total score(HADS)^a^	16.69	9.51
≤8^b^	103.00	0.30
>9^b^	236.00	0.70
Medical coping skills		
Face^a^	16.76	5.39
Avoidance^a^	18.09	4.44
Resignation^a^	13.82	3.47
Total score^a^	48.67	4.53

Data are presented as follows: a = means ± standard error (SE); b = n (%). QoL, Quality of Life; 36-item Functional Assessment of Cancer Therapy-Breast.

**Table 3:** Factors predicting scores for the QOL for breast cancer patients.

Factors	Univariate				Multivariate			
	Beta	95% CL		*p*	Beta	95% CL		*p*
		Lower	Upper			Lower	Upper	
Psychological distress								
No (DT score < 4 and HADS score ≤ 8)	ref	–	–	–	ref	–	–	–
Yes (DT score ≥ 4 or HADS score > 9)	15.936	9.574	12.270	<0.001***	−3.125	−1.285	−0.292	0.002**
Total score of DT	−40.056	−1.443	−1.308	<0.001***	1.087	−0.088	0.305	0.278
Total score of HADS	−42.405	−0.748	−0.681	<0.001***	−4.310	−0.375	−0.140	<0.001***
Body image (BIS)								
Affective Dimension	−36.212	−2.350	−2.108	<0.001***	−0.128	−0.296	0.260	0.898
Cognitive Dimension	−36.717	−2.396	−2.152	<0.001***	−0.218	−0.318	0.255	0.027*
Behavioral Dimension	−23.629	−3.815	−3.229	<0.001***	0.579	−0.210	0.385	0.047*
Total score	−39.634	−0.979	−0.886	<0.001***	−0.177	−0.261	0.218	0.038*
Medical coping skills								
Face	9.619	0.507	0.768	<0.001***	−0.124	−0.183	0.161	0.010 **
Avoidance	−16.342	−1.242	−0.975	<0.001***	−0.591	−0.225	0.121	0.555
Resignation	−10.934	−1.289	−0.896	<0.001***	0.783	−0.113	0.262	0.434
Total score	−10.155	−0.944	−0.638	<0.001***	−0.242	−0.187	0.146	0.023 *

CL, confidence limits; DT, Distress Thermometer; HADS, Hospital Anxiety and Depression Scale; ref, reference. Significance: *p < 0.05; ** *p* < 0.01, ***p < 0.001. Multivariate regression model included variables with p values < 0.10 in univariate regression analyses treated as covariates including Educational Level, Careers, Work status, Per capita monthly household income, Medical cost, Self-managed ability, Surgical form, Cancer stage, Hair Loss Types, Mode of treatment, Number of chemotherapy cycles, and Number of radiation cycles (Table 1).

## 4 Results

The characteristics of the study participants are delineated in Table 1, with an average age of 33.47 years. Among the 339 young breast cancer patients included in the study (age range: 18–40 years, mean age: 33.47 years), 2.06% reported having no children, 83.19% indicated no religious affiliation, 64.9% had attained a high school education or below, 21.24% were in active employment, 32.74% had a monthly household income below 3,000 yuan, and 75.81% were covered by medical insurance. Based on Unifactorial Analysis, we explored the associations between participant characteristics and FACT-B scores (Table 1). Significant associations were observed between FACT-B scores in young breast cancer patients and variables such as education level, occupation, employment status, average monthly household income, medical expenses, self-care ability, surgical approach, cancer stage, type of hair loss, treatment method, number of chemotherapy sessions, and number of radiotherapy sessions. Conversely, age, the number of children and the religion exhibited no significant relationship with FACT-B scores. In the subsequent multiple regression model, these significant variables were included as control covariates for the analysis of FACT-B scores (Table 3).

In terms of Quality of Life (FACT-B), breast cancer patients achieved a total score of 75.75, with the highest score observed in Breast Cancer-Specific Well-Being at 18.70. Regarding Body Image (BIS), the total score was 14.45, with the highest scores observed in the Cognitive and Affective dimensions, both at 5.74. Concerning Psychological Distress (DT and HADS), 71% of survivors had a DT score of ≥4. Anxiety and depression scores were 8.28 and 8.44, respectively, resulting in a total HADS score of 16.69. Regarding Medical Coping Skills, the total score was 48.67, with the highest score observed in the Evade dimension at 18.09 (Table 2).

Based on Univariate Analysis, the independent variables, encompassing three domains of BIS (Affective Dimension, Cognitive Dimension, Behavioral Dimension), scores from the psychological distress thermometer, two domains of HADS (Anxiety, Depression), and three dimensions of medical coping (Face, Evade, Surrender), exhibited significant associations with FACT-B scores among young breast cancer patients (Table 3). Based on multiple regression analysis, Table 3 presents the important factors predicting FACT-B scores after adjusting for covariates in young women. Significant predictors of FACT-B scores after covariate adjustment include psychological distress (*β* = −3.125; *p* = 0.002), anxiety and depression (*β* = −4.31; *p* < 0.001), total score of body image (*β* = −0.177; *p* = 0.038), cognitive dimension of body image (*β* = −0.128; *p* = 0.027), behavioral dimension (*β* = 0.579; *p* = 0.047). Total score of medical coping (*β* = −0.242; *p* = 0.023) and Face dimension of medical coping (*β* = −0.124; *p* = 0.01). After conducting multiple regression analysis, the emotional dimension of body image and the evade and surrender dimensions of medical coping were found to have no significant effects, possibly due to interference from multiple factors.

## 5 Discussion

This study rigorously explores the interrelationships between psychological distress, body image, medical coping strategies, and the overall QOL among young women diagnosed with breast cancer. Our findings reveal significant associated among these variables in young breast cancer patients. Specifically, heightened psychological distress, challenges with body image perceptions, and varying medical coping mechanisms emerge as pivotal factors influencing the QOL outcomes in this demographic. Therefore, young breast cancer patients encounter complex socio-psychological changes, necessitating personalized support interventions for psychological well-being and coping strategies throughout the entirety of their treatment and recovery processes.

### 5.1 The impact of sociodemographic factors on the QOL of YBC patients

Studying the social demographic factors influencing body image, psychological distress, and QOL among young breast cancer patients is crucial and intricate, given their multifaceted physical and psychological challenges that profoundly affect their well-being. In our research, we discovered that breast cancer patients with a primary school education tend to report higher QOL. Despite lacking higher education, they exhibit trust in doctors, high treatment compliance, and strong confidence in therapy, aligning with findings from D. Kangs’s study (Kang et al., 2022). Regarding occupational status, our study indicates that both employed and self-employed individuals tend to report higher QOL. Research by Li et al. (2024) reveals that a cancer diagnosis disrupts life plans, increases financial burdens, and complicates maintaining body image concerns. Nevertheless, continuing to work provides ongoing income, while self-employment allows sufficient time for treatment, contributing to relatively higher QOL among those with health insurance. The cancer stage, Surgical form, treatment methods, and the presence of side effects such as hair loss all influence the body image of young breast cancer patients (Rosenberg et al., 2020). Physical changes such as breast deformity, hair loss, skin alterations, and weight gain exacerbate body image concerns (Chen et al., 2012), thereby impacting the patients’ QOL. A study (Di Nardo et al., 2022) on the management of long-term side effects in breast cancer patients undergoing chemotherapy reveals that symptoms such as fatigue, insomnia, hair loss, peripheral neuropathy, cognitive impairment, estrogen deprivation, and cardiac toxicity impose significant dual burdens on patients, both physically and psychologically. Thus, the length of chemotherapy cycles strongly influences patients’ body image, psychological distress, and overall QOL. Our research findings align with this, demonstrating that shorter chemotherapy cycles correspond to higher QOL for patients.

### 5.2 The impact of body image on the QOL

Body image levels significantly affect the QOL of YBC patients. In other words, satisfaction with the visual bodily perception of oneself can lead to changes in the physical and subjective well-being of patients, permeating aspects of their QOL (Battistello et al., 2024). In our study, we noted that an elevated score in the Behavioral Inhibition System (BIS) dimension was associated with an adverse predictive influence on the QOL among young breast cancer patients which supports Hypothesis 1. Individuals exhibiting elevated scores in the behavioral dimension may employ a spectrum of detrimental strategies in response to their unease regarding their physical appearance. Such strategies may encompass extreme dietary control, immersion in excessive exercise, potential avoidance of social interactions, and a persistent pursuit of external validation (Dominici et al., 2021). These adverse strategies could result in survivors experiencing social isolation and emotional distress. This discovery is supported by previous research, suggesting that these adverse behaviors may indirectly contribute to social isolation, consequently diminishing the QOL in breast cancer patients. Moreover, these behaviors could heighten mortality risks and pose a potential threat to intestinal ecosystem balance (Hilakivi-Clarke and de Oliveira Andrade, 2023).

Furthermore, our findings indicate that lower scores in the cognitive dimension of BIS are associated with a favorable predictive impact on the QOL among young breast cancer patients, which also supports Hypothesis 1. Women with better conceptualization of their body image have been found to cope better with cancer. Poor body image perceptions have the potential to negatively impact the physical and psychological functioning of the breast cancer survivor and also the well-being of their partnered relationships. In the immediate postoperative period, it is possible that the wounds and functionality of breast cancer patients may not fully return to an optimal state. Nevertheless, as time progresses, survivors frequently demonstrate an ability to adapt to alterations in their physical appearance, indicating a more positive outlook in terms of body image perception (Brunet et al., 2022). Conversely, breast cancer treatment can leave scars and deformities in the skin and structure of the breast, potentially eliciting negative self-perception among survivors. Despite individual variations in breast cancer patients’ discontent with their body image, young breast cancer patients may undergo a decline in self-esteem and an escalation in psychological distress when grappling with anxieties about their post-treatment appearance (Rosenberg et al., 2020). Therefore, healthcare professionals should pay greater attention to the influence of body image on the psychological well-being and quality of life of young breast cancer patients.

### 5.3 The impact of psychological distress on QOL

Women believe that their emotional state is closely linked to their body image. They perceive that positive emotions enhance their body image, whereas negative emotions are associated with a negative body image (Brunet et al., 2022). Previous studies (Hodgkinson et al., 2007; Nowicki et al., 2015) have indicated that following aggressive treatment for breast cancer, young breast cancer patients may experience higher psychological distress including fear, anxiety, and depression about cancer prognosis, as well as challenges such as body image issues, sexual dysfunction, and difficulties in work and family life. These issues have the potential to impact their coping abilities and overall QOL. Our findings are partially consistent with previous research, underscoring a notable adverse effect of heightened psychological distress on the overall QOL for survivors, which supports Hypothesis 2. Under the veil of psychological distress, breast cancer patients may grapple with issues like sleep disturbances, alterations in appetite, and diminished interest in life activities. Furthermore, this could potentially result in a compromised immune system, heightening the susceptibility to illness and exacerbating symptoms of certain chronic conditions (Phoosuwan and Lundberg, 2022). Its influence transcends the emotional dimension, permeating into the realms of physiology, social dynamics, and physical well-being, thereby intensifying the burdens imposed by the illness. Studies (Wilson et al., 2022) have found that younger women’s worse psychological state after breast cancer surgery may contribute to worsened pain outcomes. Therefore, considering the psychological impact on young breast cancer patients, proactive psychological screening and comprehensive mental health resources are essential. Furthermore, interdisciplinary collaboration (including neurology, nutrition, and cosmetic surgery disciplines) to help young breast cancer patients mitigate the physical and psychological toll of treatment and enhance their quality of life is crucial.

### 5.4 The impact of medical coping on QOL

This research reveals that young breast cancer patients tend to employ the facing coping mode in navigating their illness, all of which exert positive influences on their overall QOL. This finding aligns with the results of Chen’s research (Chen et al., 2020) and also supports Hypothesis 3. Coping strategies denote the actions and measures individuals employ when confronted with challenges or stressful events, commonly distinguished into two dimensions: positive coping and negative coping (Feifel et al., 1987). Among them, “confrontation” is considered a positive coping strategy, fostering enhanced satisfaction, alleviated psychological stress, and a diminished risk of disease exacerbation for breast cancer patients (Yamani Ardakani et al., 2020). This proactive engagement attitude contributes to improving patients’ QOL, empowering them to approach all facets of life with a more positive outlook (Schapira et al., 2022). Just like young breast cancer patients who choose to face their challenges with positivity, they have learned to accept and show compassion towards their bodies after experiencing different stages in life. They have also developed a better understanding of and greater tolerance for their imperfections (Brunet et al., 2022). However, “avoidance” and “resignation” are typically regarded as negative coping methods. It is notable that a significant proportion of patients in this study tended to choose “avoidance” as their primary coping strategy. Within the context of this study, “avoidance” is construed as a proactive coping approach. A study indicated that employing an avoidance coping strategy could alleviate survivors’ anxiety and depressive emotions, fostering a positive influence on the outcomes of problem-solving (Buffart et al., 2020). Furthermore, research substantiates that “avoidance” serves as a self-protective strategy, enabling survivors to temporarily distance themselves from the repercussions of the illness, addressing disease-related issues when their emotional resilience is better established (Straight et al., 2019). Therefore, clinicians should carefully consider various psychosocial and cultural factors that may influence coping behaviors and integrate interventions to foster more adaptive coping strategies. Implementing psychological support, educational programs, and mindfulness-based interventions (Shao et al., 2020) can assist patients in developing acceptance and resilience, potentially mitigating psychological distress, body image concerns, and their impact on the QOL among young breast cancer patients (Van Helmondt et al., 2023). The facing coping pattern in clinical practice holds promise for enhancing overall QOL for young breast cancer patients. Further research is necessary to explore the mechanisms linking breast cancer patients’ coping and avoidance coping patterns with QOL.

### 5.5 Limitations

We acknowledge the limitations of our study, given that participants were exclusively from Wuhan, which may limit the generalizability of our findings. Future research should involve diverse populations to verify applicability across different regions. Furthermore, considering the dynamic nature of quality of life and the cross-sectional design of our study, we plan to conduct longitudinal research. This will explore the evolution of body image, psychological distress, coping strategies, and their impact on the quality of life of breast cancer patients, with the goal of enhancing intervention effectiveness. Finally, it’s important to note that several psychological factors influence the quality of life of breast cancer patients, including not only psychological distress, anxiety, depression, and body image, but also fear of disease recurrence and patient resilience. These factors can be further explored in future research.

## 6 Conclusion

This study validates the profound influence of psychological distress, body image, and medical coping strategies on the QOL of young breast cancer survivors. Lower levels of psychological distress, diminished body image scores, and the embrace of constructive medical coping strategies emerge as robust predictors of Enhanced QOL among this demographic. These discoveries hold promise for enriching health education and promotional initiatives tailored to young breast cancer survivors, empowering healthcare teams to more adeptly address and fulfill their psychological well-being requirements. Consequently, these revelations are poised to inform forthcoming intervention strategies, thus holistically advancing treatment efficacy and elevating overall QOL.

## Data availability statement

The datasets utilized and/or analyzed in this study are available upon reasonable request from the corresponding author. Requests to access the datasets should be directed to zn001737@whu.edu.cn.

## Ethics statement

The studies involving humans were approved by Ethics Committee of Zhongnan Hospital of Wuhan University. The studies were conducted in accordance with the local legislation and institutional requirements. The participants provided their written informed consent to participate in this study. Written informed consent was obtained from the individual(s) for the publication of any potentially identifiable images or data included in this article.

## Author contributions

HY: Data curation, Formal analysis, Software, Validation, Writing – original draft. MX: Conceptualization, Data curation, Methodology, Writing – original draft. YC: Data curation, Formal analysis, Visualization, Writing – review & editing. QZ: Project administration, Writing – review & editing, Data curation, Investigation, Visualization. YL: Data curation, Writing – review & editing, Supervision. XD: Writing – review & editing, Funding acquisition, Project administration, Resources. CZ: Funding acquisition, Project administration, Resources, Writing – review & editing.
